# Effects of Proteins and Mineral Ions on the Physicochemical Properties of 1,3-Dioleoyl-2-Palmitoylglycerol Emulsion to Mimic a Liquid Infant Formula

**DOI:** 10.3389/fnut.2022.808351

**Published:** 2022-06-13

**Authors:** Qi Wang, Yuxi Xu, Yanchen Liu, Fang Qian, Guangqing Mu, Xuemei Zhu

**Affiliations:** ^1^School of Food Science and Technology, Dalian Polytechnic University, Dalian, China; ^2^State Key Lab of Food Science and Technology, Nanchang University, Nanchang, China

**Keywords:** calcium, potassium, OPO emulsion, stability, lipid oxidation

## Abstract

Proteins and minerals in infant formula not only serve as nutrients, but also have important effects on the physical and chemical stability of emulsions. In this study, calcium carbonate (0 or 9.08 mM) and potassium chloride (0 or 15.96 mM), as representatives of divalent and monovalent minerals, were added to 1,3-dioleoyl-2-palmitoylglycerol (OPO) emulsions in different ratios (10:0, 9:1, 6:4, 5:5, and 0:10) of whey protein isolate (WPI) and sodium caseinate (CN). The influence of proteins and minerals on emulsion stability was investigated by analyzing particle size, zeta potential, creaming index, rheological properties, storage stability, and lipid oxidation. 1,3-dioleoyl-2-palmitoylglycerol (OPO) emulsions could be destabilized by adding Ca^2+^, as shown by the increase in particle size index, creaming index, and the decrease in zeta potential magnitude. Divalent ions could affect the electrostatic interactions between lipid droplets and the interactive effects of ion surface adsorption. In addition, the effect of different protein ratios on the physical stability of emulsions was not significant under the same ion-type conditions. In terms of chemical stability, higher oxidized values were found in emulsions stabilized with only CN than in those containing WPI. Our study showed that protein ratios and minerals played an important role in the stability of OPO emulsions, which might provide a reference for the development and utilization of liquid infant formula.

## Introduction

In the first 6 months of life, breast milk from healthy and well-nourished mothers is regarded as the best food for infants (1). In addition, it contains nutrients that aid in the protection and maturation of the infant intestine (2). Breast milk contains 3–5% fat, 0.8–0.9% protein, 6.9–7.2% carbohydrate, and 0.2% minerals, and other physiologically active components (IgA, bifidus factor, etc.) (3). Among the macronutrients, breast milk lipids are the primary source of energy and vital nutrients for the newborn. In terms of the composition of breast milk fat, triglycerides account for 98% of the fat content and oleic acid, palmitic acid, and linoleic acid are the three most abundant fatty acids, followed by stearic and myristic acids, where 70% of palmitic acid is esterified at the sn-2 position and unsaturated fatty acids (oleic acid, linoleic acid, etc.) are located at the sn-1,3 position. The unique composition and distribution of fatty acids leads to a representative human milk fat named 1,3-dioleoyl-2-palmitoylglycerol (OPO) (4, 5).

Human milk fat substitutes refer to the triglyceride mixture synthesized by modern enzymatic modification technology. It modifies animal and vegetable fats according to the nutritional composition, fatty acid composition, and fat distribution of human milk (6, 7). It is intended to meet the nutritional needs of infants and their growth and development. Carnielli et al. provided solid evidence that breastfed newborns absorbed palmitic acid at the sn-2 position (8). López-Lópe et al. compared the total fatty acid compositions with the sn-2 positional fatty acid compositions of colostrum, transitional milk, mature milk, and infant formula. They found that infant formula containing sn-2 positional palmitic acid is more easily absorbed than regular formula (9). Koo et al. demonstrated that palmitic acid in the sn-2 position of infant formula had a physiological function, including enhancing calcium absorption in the small intestine and decreasing bone mass (10). OPO, as a triglyceride typical of breast fat, influences infant digestion and absorption, making OPO a value-added ingredient in commercial powder infant milk.

Currently, liquid infant formula is a potential popular product because it is easier to drink and has brighter packaging that appeals to infants and young children. For the development of liquid infant formula products, the physical and chemical stability of these products are just as crucial as their nutritional content. The emulsifier has a significant impact on emulsion stability. As main milk proteins, whey protein (WP) and casein are important emulsifiers in liquid infant formula products, which have also been widely used in the food industry (11). The ratio of WP to casein in breast milk varied depending on the stage of breastfeeding, for example, it could be as high as 9:1 in early lactation and subsequently decline to 5:5 in late lactation. An overall ratio of roughly 6:4 is used in most infant formula products. Milk proteins adsorb at the interface between the two phases of the oil-in-water (O/W) system, forming a protective layer on the surface of the lipid droplet before and after homogenization and inhibiting interfacial tension to maintain emulsions. It is well known that WP and casein have different properties, which have an impact on the physical and chemical stability of liquid infant formula.

Minerals in infant formula have a significant impact on the physical and chemical stability of emulsions, in addition to the complex fat structure and protein composition. Minerals boost the ionic strength in the aqueous phase, lowering the electrostatic repulsion between droplets and promoting phase separation (12–15). Some minerals bind to oppositely charged groups on the surface of emulsion droplets, decreasing their zeta potential and thus reducing electrostatic repulsion (16). In several commercial emulsions, calcium-induced flocculation and creaming have been identified as the sources of long-term destabilization (17).

Previous studies have shown that the creaming stability of emulsions with 0.5% protein decreased, while the emulsion with 3% protein was not affected by the addition of CaCl_2_ to 0.5% (18, 19). In this study, an OPO emulsion was prepared to imitate liquid infant formula. We investigated the effect of a series of protein ratios (10:0, 9:1, 6:4, 5:5, and 0:10) and ion types on the physicochemical properties of the OPO emulsion. The ratios were selected based on the content of WP and casein in breast milk throughout the lactation period and the combined effect of monovalent (represented by K^+^) and divalent (represented by Ca^2+^) ions in infant formula milk powder. This study may provide a theoretical basis for the physical stability of liquid infant formula.

## Materials and Methods

### Materials

1, 3-dioleic-2-palmitate triglyceride-type human milk fat substitutes (triglycerides > 98%) were kindly donated by Renzhichu Technology Group Ltd. (Jiangxi, China), which would be used directly for producing infant formula. Provon® whey protein isolates (WPI, purity 90%) were purchased from Glanbia Nutritionals Ltd. (Ireland). Sodium caseinate (CN, BR, from bovine milk) was purchased from Macklin Biochemical Co., Ltd. (Shanghai, China). Other solvents and reagents were of analytical grade.

### Preparation of O/W Emulsions

Oil-in-water emulsions were prepared according to previous reports with simple modifications. Briefly, an aqueous solution consisting of WPI: CN (1% w/v in total) and phosphate buffered saline (PBS, 0.01 M, pH 7.0) was stirred for 5 h to ensure complete dissolution of the protein. After storing the emulsion at 4°C overnight, Ca^2+^ and K^+^concentrations (9.08 and 15.96 mM) were added once the protein dispersion had returned to room temperature. The solutions were stirred for 10 min using a DS-101S magnetic stirring apparatus (Yarong Instrument Ltd., Zhengzhou, China) to ensure complete dissolution of the salts. To make a fresh emulsion, 10% (v/v) of OPO human milk fat substitutes were mixed with sufficient dissolution at 12,000 rpm for 3 min, homogenized 2 times at 30 MPa, and loaded with sodium azide (0.02% w/v) as an antibacterial agent. The pH of the emulsion was adjusted to 7.0 with 1 M NaOH and/or 1 M HCl for analysis.

### Average Particle Size and Zeta Potential

The particle size distributions and zeta potential of infant formula emulsion samples were measured using a nano-ZSE laser particle size analyzer (Malvern Instruments Ltd., England) according to dynamic light scattering. Infant formula (×100 dilution) emulsion samples were prepared to avoid multiple scattering effects, and the absorption of 0.01 was used to calculate particle size distributions as described previously (20). Each sample was tested three times, and the data are presented as average ± standard deviation (SD).

### Creaming Index

The infant formula emulsion sample (pH 7.0) was transferred to a test tube [internal diameter (Φ) = 20 mm, height (H) = 500 mm], and then stored at room temperature for 4 months. After storage, several samples were separated into a thin “cream” layer at the top and a transparent “serum” layer at the bottom (21). The creaming destabilization kinetics was evaluated by measuring creaming index as the percentage of serum layer height (*H*_*S*_) from the total emulsion height (*H*_*T*_) (22).


$$\begin{array}{l} Creaming\;index=\frac{H_{S}}{H_{T}}\times 100\% \end{array}$$

### Rheological Properties

Rheological properties were carried out in a DHR-2 rheometer (TA Instruments Ltd., New Castle, DE, USA). The temperature was maintained at 25 ± 0.5°C. The apparent viscosity of the emulsion sample was measured upon shear rate ramp-up from 5 to 100 s^−1^ according to the modified method as described previously (23). The exposed surfaces of the samples were covered with a thin layer of silicone oil to prevent dehydration. The shear rate was then recorded as the shear stress was increased.

### Storage Stability

The infant formula emulsion sample (2 ml) was poured into a cuvette and then stored at room temperature for 40 days, and 5 μl of each sample was taken out at the time intervals of 0, 8, 16, 24, 32, and 40 days for analysis. The physical stability of emulsions during storage was characterized by regular sampling and measuring their average particle size and zeta potential.

### Lipid Oxidation

The method for characterizing the oxidation stability of oil is based on the method of Qiu et al. (24) and slightly modified. The emulsion sample was placed in an oven at 60°C, and the samples were taken out at different time periods (0, 10, 20, 30, and 40 days) for the analysis of oxidation state.

#### Primary Oxidation Product Hydrogen Peroxide Value

Primary oxidation product hydrogen peroxide value (POV) was measured according to the report of Zeng et al. (25) with slight modifications. Briefly, 1 ml of the emulsion sample was vortexed vigorously three times with 5 ml of isooctane: isopropanol (2:1 v/v) followed by centrifugation for 5 min at 2,800 × g (FA-45-6-30 rotor, centrifuge 5804R, Eppendorf AG, Hamburg, Germany). Then, 0.2 ml of the isooctane: isopropanol extract was mixed with 4.8 ml of methanol: butanol (2:1 v/v). This mixture was then reacted with 20 μl of 3.94 M potassium thiocyanate and 20 μl of ferrous iron solution. The mixture was allowed to react for 20 min at room temperature in the dark before measuring the absorbance of the sample at 510 nm using a TU-1900 spectrophotometer (Baiqi Biotechnology Ltd., Jinan, China). The concentration of hydroperoxides was determined based on a standard curve of hydroperoxide.

#### Thiobarbituric Acid Reactive Substances

Thiobarbituric acid reactive substances (TBARS) were measured according to Xu et al. (26). The infant formula emulsion sample (0.6 ml) was combined with 4.0 ml of TBA (thiobarbituric acid) solution (prepared by mixing 15 g of trichloroacetic acid, 0.375 g of TBA, 1.76 ml of 12 M HCl, and 82.9 ml of H_2_O) in test tubes and placed in a boiling water bath for 30 min. The tubes were cooled to room temperature for 10 min and then centrifuged at 860 × g (2,500 rpm) for 20 min followed by measuring the absorbance at 532 nm. TBARS were calculated from a standard curve prepared with 1,1,3,3-tetraethoxypropane.

### Statistical Analysis

The results are presented as means ± SD using SPSS 18.0 software (IBM Corporation, NY, USA) and Origin 85 software (Microsoft Corporation, Redmond, WA, USA). The statistical analysis was performed by one-way ANOVA followed by Duncan's test at *p* < 0.05.

## Results

### Particle Size and Zeta Potential Analysis

**Figure 1:**
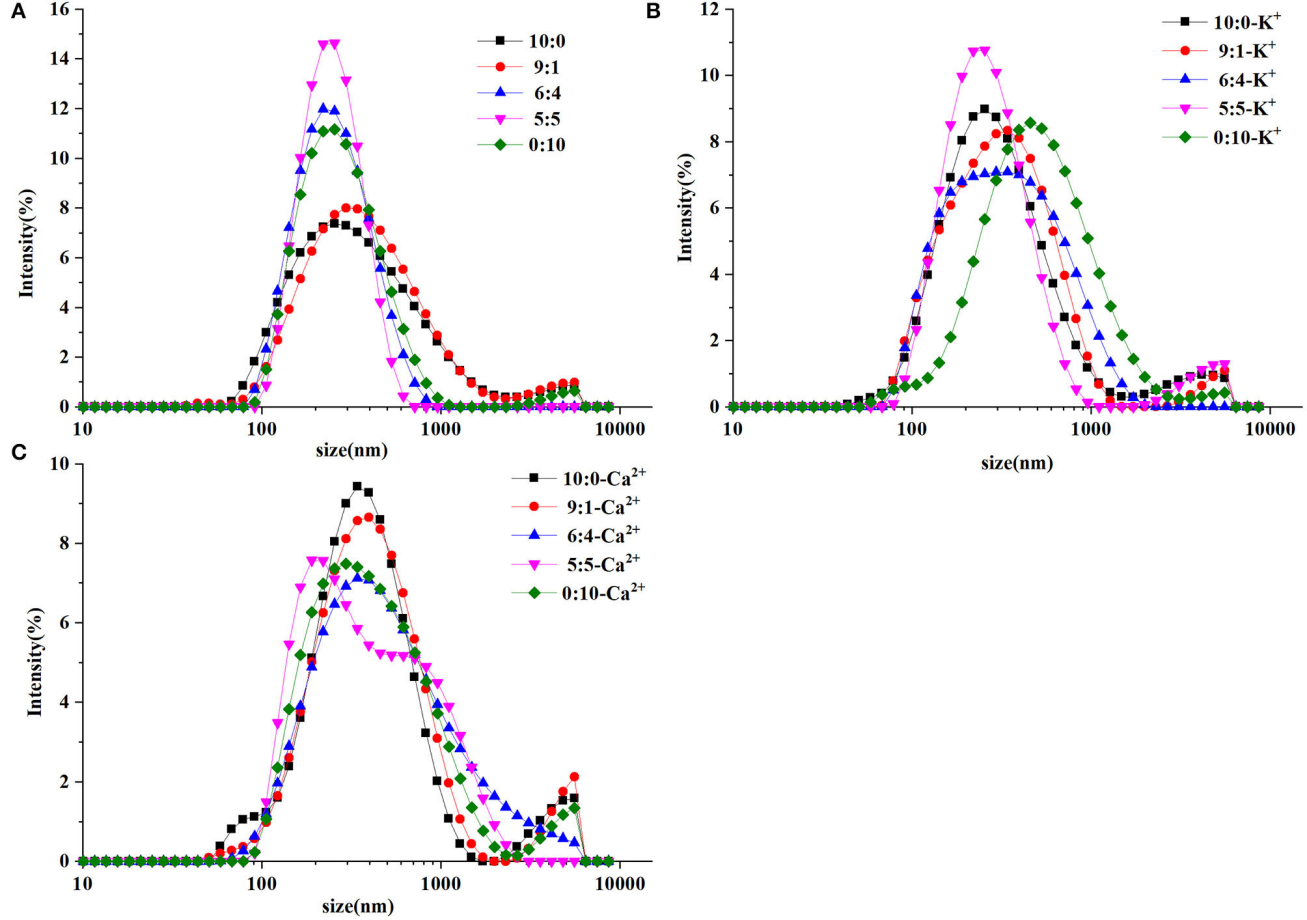
Particle size distribution of control **(A)**, containing K^+^ **(B)**, and Ca^2+^ **(C)** of 1,3-dioleoyl-2-palmitoylglycerol (OPO) emulsions (10% v/v OPO human milk fat substitutes) with different ratios of whey protein isolate (WPI) and caseinate (CN; 10:0, 9:1, 6:4, 5:5, 0:10, 1% w/v in total) as emulsifiers.

Particle size distribution in the OPO emulsion samples is illustrated in Figure 1. In Figure 1A, the emulsion in the control (without ions) with a higher WPI ratio (10:0 and 9:1) was characterized with larger particles and a tail peak in peak distribution. The result indicated that a number of original droplets had accumulated. Emulsions with increasing CN ratios (6:4, 5:5, and 0:10) showed a single peak distribution and a tight particle size distribution, indicating a good symmetry dispersion. Compared with the control, the particle size distribution of the ion groups showed a bimodal distribution. The size distribution was control group < K^+^ group < Ca^2+^ group, indicating that the large particles in emulsions were gradually increased and formed by droplet aggregation.

**Figure 2:**
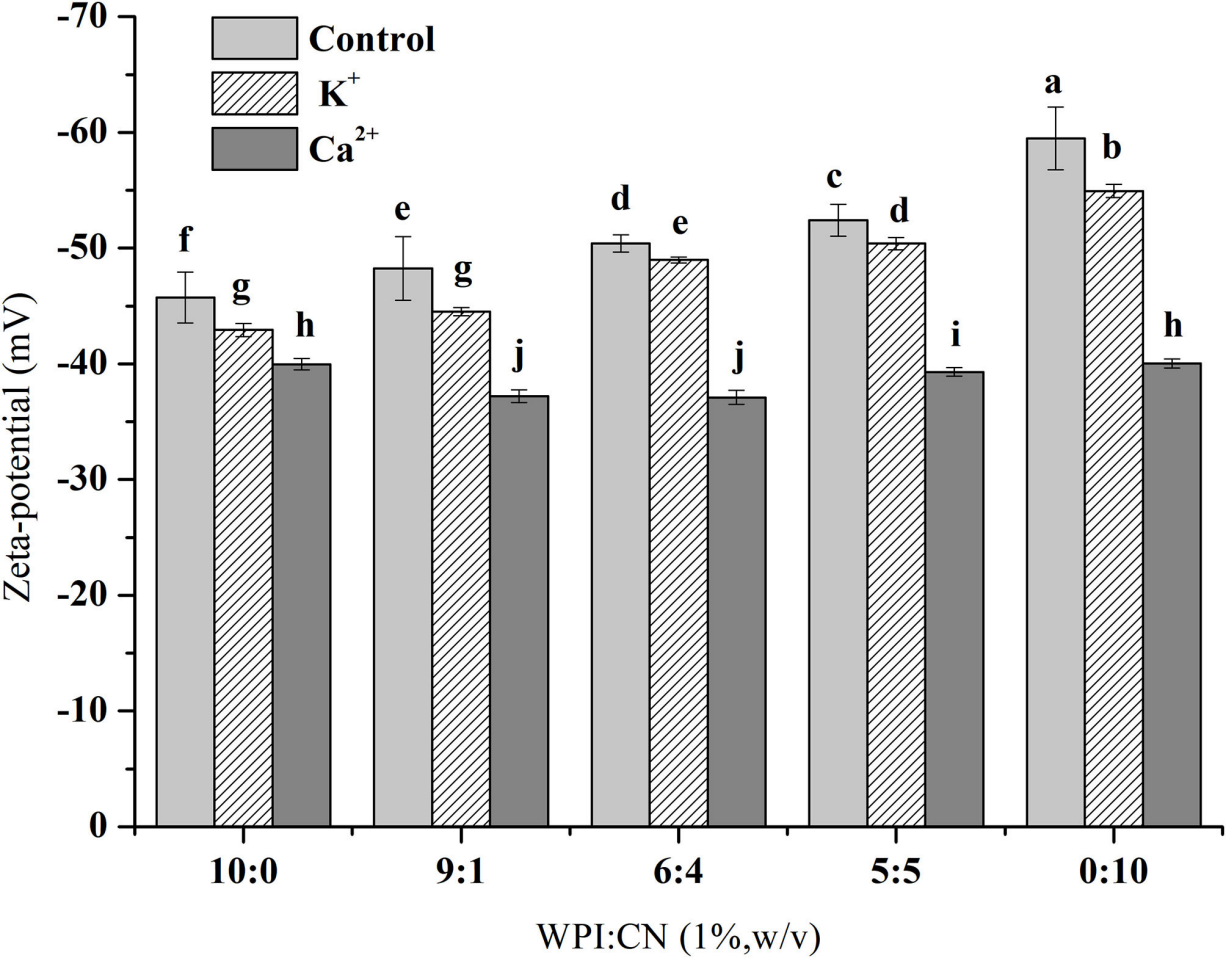
Zeta potential of fresh OPO emulsions (10% v/v OPO human milk fat substitutes) with different ratios of WPI and CN (10:0, 9:1, 6:4, 5:5, 0:10, 1% w/v in total) as emulsifiers. The results are expressed as mean ± SD (*n* = 3). Different letters (a–j) indicate significant differences at the 0.05 level.

The zeta potential values of emulsions with various WPI and CN ratios are shown in Figure 2. The zeta potential amplitude of the control samples (without ionic addition) decreased as the CN concentration in emulsions increased. When adding K^+^ and Ca^2+^ into emulsions, the absolute zeta potential value did not change significantly with increasing CN ratio. Under the conditions of different or same protein ratios, the absolute zeta potential value was K^+^ group > Ca^2+^ group and the change in the absolute zeta potential value was Ca^2+^ group > K^+^ group > control group.

### Creaming Index During Storage

According to Table 1, the impacts of different protein ratios and mineral ions in emulsions did not show layering behavior when emulsions were placed at room temperature for 20 days, except the 6:4 and 5:5 (WPI:CN) emulsions after adding K^+^ and Ca^2+^, which showed a slight alteration (7.92 ± 0.71, 13.59 ± 0.43, 10.79 ± 2.76, and 7.13 ± 1.24%, respectively). However, varying degrees of cream layer separation were found from 20 to 120 days. Emulsions emulsified by WPI were stable, whereas emulsions with K^+^ and Ca^2+^ showed the most obvious layering phenomenon (81.69 ± 1.84 and 82.02 ± 6.44%). It is shown that the overabundance of unabsorbed biopolymers resulted in higher droplet accumulation, larger particles, and more pronounced stratification behavior after longer storage time.

**Table 1:** The creaming index of fresh 1,3-dioleoyl-2-palmitoylglycerol (OPO) emulsions (10% v/v OPO human milk fat substitutes and whey protein isolate (WPI): CN/10:0, 9:1, 6:4, 5:5, 0:10, 1% w/v in total) for 120 days.

**WPI: CN** **(1%, w/v)**	**20 days**	**40 days**	**60 days**	**120 days**
10:0	ND	ND	ND	0.78 ± 0.41
9:1	ND	ND	3.72 ± 0.59^b^	26.71 ± 5.14^a^
6:4	ND	2.90 ± 0.16^b^	8.04 ± 1.08^b^	40.34 ± 2.73^a^
5:5	ND	5.05 ± 2.02^b^	7.07 ± 1.00^b^	43.60 ± 1.34^a^
0:10	ND	3.80 ± 0.94^c^	6.63 ± 0.09^b^	38.15 ± 5.62^a^
10:0-K^+^	ND	ND	ND	1.96 ± 0.13
9:1-K^+^	ND	1.55 ± 0.43^c^	7.01 ± 3.98^a^	13.04 ± 1.94^a^
6:4-K^+^	7.92 ± 0.71^c^	6.38 ± 2.06^c^	39.98± 2.46^b^	71.84 ± 6.63^a^
5:5-K^+^	13.59 ± 0.43^c^	10.58 ± 6.05^c^	28.95 ± 4.34^b^	65.23 ± 7.11^a^
0:10-K^+^	ND	21.81 ± 4.71^c^	77.99 ± 4.02^b^	81.69 ± 1.84^a^
10:0-Ca^2+^	ND	ND	ND	2.49 ± 0.42
9:1-Ca^2+^	ND	3.67 ± 2.07^c^	5.39 ± 4.06^b^	23.43 ± 10.28^a^
6:4-Ca^2+^	10.79 ± 2.76^d^	18.27 ± 5.00^c^	31.33 ± 4.66^b^	64.92 ± 6.32^a^
5:5-Ca^2+^	7.13 ± 1.24^d^	16.51 ± 3.36^c^	32.11 ± 1.42^b^	72.58 ± 5.56^a^
0:10-Ca^2+^	ND	16.09 ± 2.30^c^	78.03 ± 4.64^b^	82.02 ± 6.44^a^

a−d *Different small letters represent the significance of the difference (p < 0.05); ND means not detected*.

### Viscosity Analysis

**Figure 3:**
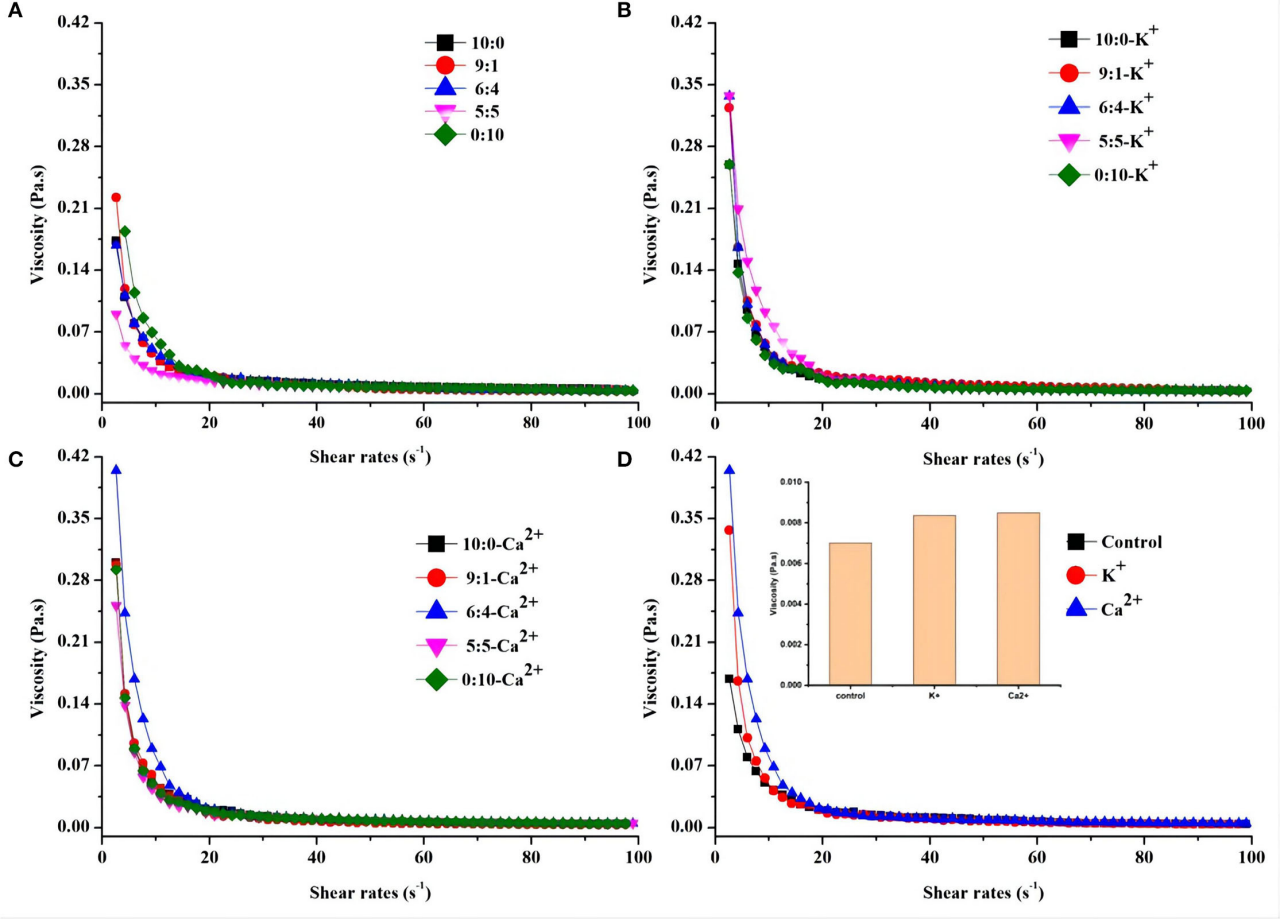
Shear stress and viscosity relationships of OPO emulsions (10% v/v OPO human milk fat substitutes) at different protein ratios (WPI:CN/10:0, 9:1, 6:4, 5:5 and 0:10, 1% w/v in total; **(A)** either in the presence of K^+^ **(B)** or Ca^2+^ **(C)**. **(D)** Comparison of the shear stress and viscosity relationship of OPO emulsions for WPI:CN/6:4 (control) with K^+^ or Ca^2+^.

The rheological behavior of the OPO emulsion system was examined as a function of different protein ratios and ionic strength. In particular, the flow behavior of the emulsion sample was investigated taking into account the dependence of the shear stress on the sample viscosity. The effect of different protein ratios and ionic strength on the rheological properties of emulsions was examined (Figures 3A–D). The flocculated emulsions exhibited strong shear thinning behavior (Figure 3). Viscosity decreased with increasing shear rate. When the shear rate was in the range of 5–10 s^−1^, the viscosity of each emulsion group decreased rapidly as shear stress increased. Then, the viscosity tended to be consistent within the shear rate of 20–100 s^−1^. Compared with Figure 3A, the viscosity of the K^+^ and Ca^2+^ groups was higher than the control group in the range of 5–10 s^−1^. This difference could be attributed to factors such as flocculated volume fraction of the droplets, flocculation size, and spatial distribution or intensity of interaction between flocculated droplets (27). Different protein ratios were found to be insensitive to changes in emulsion viscosity.

To understand the relationship between shear stress and viscosity of emulsions by the addition of K^+^ and Ca^2+^, only the ratio of 6:4 (WPI:CN) was chosen for viscosity analysis. Figure 3D shows that the viscosity of the 6:4 emulsion without salt ions is lower than that of the emulsion with K^+^ and Ca^2+^, which was Ca^2+^ group > K^+^ group > control group in the range of 5–20 s^−1^.

### Physical Stability of Emulsions During Storage

The storage stability (particle size and zeta potential) of emulsions after 40 days is shown in Table 2. The results showed that the emulsion particle size gradually increased with storage time. It was also demonstrated that the absolute value of the zeta potential gradually decreased as the emulsion storage time increased. In detail, the particle size of emulsions without ions and with K^+^ or Ca^2+^ were 355–460, 426–507, and 424–514 nm, respectively, after storage for 40 days. In addition, the zeta potential in the control, K^+^, and Ca^2+^ groups was −41.53~-51.27, −39.53~-52.33, and −29.67~-36.10 mV, respectively, after storage for 40 days. According to the analysis with or without K^+^ and Ca^2+^, the trend of the physical stability of emulsions during storage decreased in the following order: control group > K^+^ group > Ca^2+^ group.

**Table 2:** Effects of K^+^ and Ca^2+^ on storage stability (particle size and zeta potential) in different OPO emulsions (10% v/v OPO human milk fat substitutes and WPI: CN/10:0, 9:1, 6:4, 5:5, 0:10, 1% w/v in total).

**WPI: CN** **(1%, w/v)**	**0 day**	**8 days**	**16 days**	**24 days**	**32 days**	**40 days**
**Particle size**						
10:0	276.57 ± 3.29^c^	253.10 ± 1.87^d^	285.93 ± 1.50^c^	330.33 ± 13.65^b^	351.80 ± 4.65^a^	355.87 ± 3.50^a^
9:1	307.43 ± 6.02^d^	344.27 ± 3.37^c^	347.37 ± 4.22^c^	373.30 ± 4.55^b^	429.23 ± 3.34^a^	435.00 ± 2.82^a^
6:4	233.70 ± 3.22^f^	342.83 ± 0.81^e^	379.07 ± 5.05^d^	403.37 ± 5.77^c^	443.77 ± 3.77^b^	467.70 ± 1.85^a^
5:5	229.10 ± 1.18^d^	322.37 ± 2.81^c^	365.60 ± 2.50^b^	380.00 ± 16.10^b^	402.97 ± 7.11^a^	409.70 ± 9.26^a^
0:10	253.27 ± 1.68^d^	253.47 ± 2.17^d^	311.27 ± 3.02^c^	335.17 ± 3.60^b^	348.80 ± 0.46^b^	395.23 ± 4.77^a^
10:0-K^+^	259.30 ± 2.26^e^	269.77 ± 2.61^e^	329.93 ± 7.97^d^	353.17 ± 3.52^c^	383.90 ± 5.84^b^	429.93 ± 6.08^a^
9:1-K^+^	265.77 ± 1.04^e^	273.40 ± 3.15^e^	356.60 ± 4.08^d^	386.03 ± 4.66^c^	403.40 ± 3.22^b^	426.60 ± 4.50^a^
6:4-K^+^	266.80 ± 1.84^d^	254.07 ± 2.95^e^	347.70 ± 3.76^c^	363.83 ± 2.91^c^	426.60 ± 9.28^b^	452.83 ± 10.07^a^
5:5-K^+^	250.20 ± 2.91^e^	288.90 ± 4.03^d^	343.33 ± 5.09^c^	358.33 ± 8.74^c^	424.17 ± 0.81^b^	487.13 ± 3.81^a^
0:10-K^+^	281.33 ± 7.69^e^	339.77 ± 1.44^d^	381.67 ± 8.52^c^	423.33 ± 2.89^c^	469.17 ± 10.08^b^	507.30 ± 1.65^a^
10:0-Ca^2+^	317.97 ± 6.06^d^	331.27 ± 3.18^c^	354.60 ± 5.81^c^	387.17 ± 3.00^b^	413.73 ± 5.05^a^	424.33 ± 2.16^a^
9:1-Ca^2+^	355.40 ± 4.16^c^	343.17 ± 6.62^d^	369.13 ± 3.17^c^	451.83 ± 13.27^b^	464.30 ± 4.11^b^	491.93 ± 2.53^a^
6:4-Ca^2+^	361.47 ± 5.70^e^	397.30 ± 2.46^d^	454.67 ± 3.45^c^	479.07 ± 2.80^b^	486.73 ± 20.76^b^	514.10 ± 1.74^a^
5:5-Ca^2+^	304.13 ± 3.30^e^	380.60 ± 6.31^d^	408.60 ± 2.96^c^	418.67 ± 12.17^b^	427.2 ± 18.76^b^	500.90 ± 13.00^a^
0:10-Ca^2+^	352.63 ± 9.18^d^	363.90 ± 7.20^c^	376.53 ± 12.08^c^	462.50 ± 4.52^b^	465.17 ± 3.36^a^	460.90 ± 14.30^b^
**Zeta potential**						
10:0	−45.73 ± 2.21^a^	−43.87 ± 2.80^b^	−43.47 ± 2.90^b^	−42.43 ± 0.21^c^	−42.20 ± 0.26^c^	−41.53 ± 1.00^d^
9:1	−48.23 ± 2.75^a^	−45.10 ± 0.26^b^	−44.70 ± 2.35^c^	−43.47 ± 0.29^d^	−43.03 ± 0.85^d^	−42.43 ± 1.65^e^
6:4	−50.40 ± 0.75^a^	−50.10 ± 0.69^a^	−47.83 ± 0.45^b^	−47.73 ± 0.99^b^	−47.07 ± 2.66^b^	−45.67 ± 1.30^c^
5:5	−52.40 ± 1.37^a^	−52.07 ± 3.99^a^	−48.47 ± 0.32^b^	−47.13 ± 0.68^c^	−46.43 ± 0.35^d^	−46.23 ± 1.16^d^
0:10	−59.47 ± 2.72^a^	−56.77 ± 3.17^b^	−56.43 ± 0.87^b^	−52.13 ± 0.38^c^	−51.63 ± 1.40^d^	−51.27 ± 4.30^d^
10:0-K^+^	−42.93 ± 0.57^a^	−41.37 ± 0.93^b^	−41.27 ± 0.12^b^	−40.10 ± 0.61^c^	−39.57 ± 0.49^d^	−39.53 ± 0.59^d^
9:1-K^+^	−44.50 ± 0.35^a^	−44.27 ± 0.31^a^	−42.93 ± 0.82^b^	−41.50 ± 0.23^c^	−41.17 ± 0.40°	−40.87 ± 0.72^c^
6:4-K^+^	−48.97 ± 0.25^a^	−48.30 ± 0.70^a^	−47.07 ± 1.10^b^	−46.03 ± 0.31^c^	−45.07 ± 1.00^d^	−43.97 ± 0.91^e^
5:5-K^+^	−50.40 ± 0.53^a^	−50.07 ± 0.42	−47.03 ± 0.45	−46.37 ± 0.12	−46.00 ± 0.75	−45.97 ± 0.40
0:10-K^+^	−54.93 ± 0.57^b^	−56.83 ± 0.06^a^	−53.90 ± 0.70^c^	−53.17 ± 0.80^c^	−52.37 ± 0.49^d^	−52.33 ± 0.66^d^
10:0-Ca^2+^	−39.97 ± 0.50^a^	−34.63 ± 0.72^b^	−34.20 ± 0.36^b^	−33.73 ± 0.75^c^	−32.90 ± 0.21^d^	−30.47 ± 0.52^e^
9:1-Ca^2+^	−37.20 ± 0.56^a^	−37.13 ± 0.67^a^	−35.53 ± 0.68^b^	−35.13 ± 0.61^b^	−34.63 ± 0.55^c^	−31.77 ± 0.60^d^
6:4-Ca^2+^	−37.10 ± 0.62^a^	−36.80 ± 0.44	−36.63 ± 0.66	−35.90 ± 0.00	−35.70 ± 0.50	−34.90 ± 0.78
5:5-Ca^2+^	−39.30 ± 0.36^a^	−37.80 ± 0.62^b^	−37.40 ± 0.87^b^	−36.80 ± 1.10^c^	−36.10 ± 0.10^c^	−29.67 ± 0.40^d^
0:10-Ca^2+^	−40.03 ± 0.40^a^	−38.73 ± 0.74^b^	−37.67 ± 0.55^c^	−37.37 ± 0.50^c^	−37.17 ± 0.80^c^	−36.10 ± 0.61^d^

a−f *Different small letters represent the significance of the difference (p < 0.05)*.

### Lipid Oxidation of Emulsions

**Figure 4:**
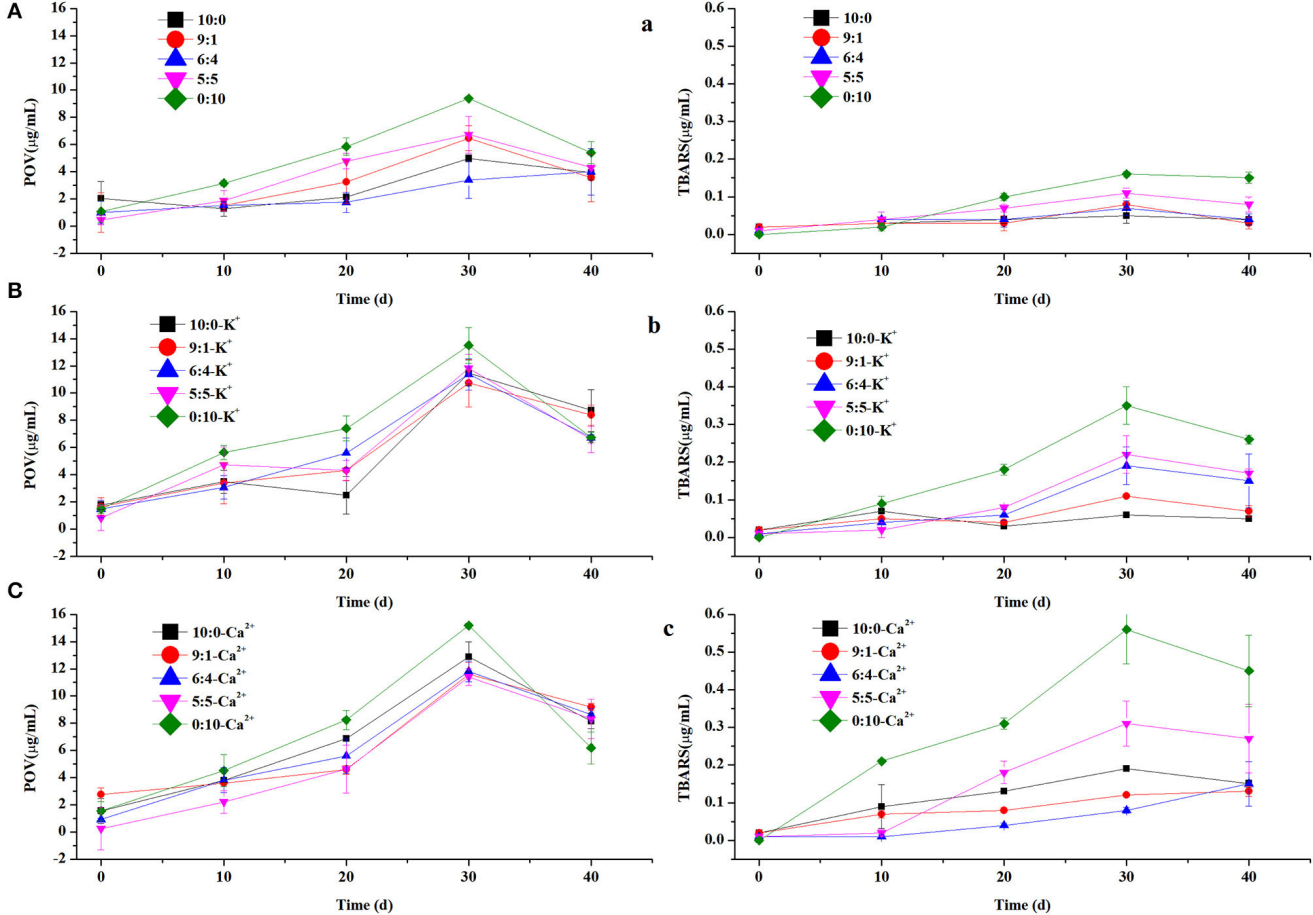
The peroxidevalue (POV) and thiobarbituric acid reactive substances (TBARS) of control, containing K^+^ **(A–C)** and containing Ca^2+^ **(a–c)** in OPO emulsions (10% v/v OPO human milk fat substitutes and WPI: CN/10:0, 9:1, 6:4, 5:5, 0:10, 1% w/v in total) as emulsifiers.

In this study, the rate of lipid oxidation in different emulsions containing K^+^ or Ca^2+^ was measured. All samples were kept at 60°C for 40 days to accelerate lipid oxidation. To investigate the rate of lipid oxidation, primary and secondary lipid oxidation products were measured as POV and TBARS (Figure 4), respectively. The results showed that the rate of lipid oxidation increased at first and then decreased as the storage time was prolonged. Due to its more compact structure and lack of phosphate groups, WPI might limit its iron-binding ability and thus promote oxidation (28). In addition, the relative oxidation value of emulsions with CN alone as the emulsifier was greater than that of other emulsions with varied ratios of WPI, based on the POV and TBARS content in Figure 4. The higher oxidation rate might be because the affinity of phosphate groups of casein molecules for iron was better than that of carboxylic acid groups of other proteins (29). In the 30-day oxidation stage, the POV content at different protein ratios (10:0, 9:1, 6:4, 5:5, and 0:10) without and with K^+^ and Ca^2+^ was 3.39–9.39, 10.73–13.52, and 11.41–15.18 μg/ml and the TBARS content was 0.05–0.16, 0.06–0.35, and 0.08–0.56 μg/ml, respectively. These data indicated that K^+^ and Ca^2+^ can promote the oxidation of emulsions, possibly because these ions destabilize the physical state of emulsions as mentioned above.

## Discussion

Whey protein isolate and CN are typical protein-type emulsifiers, which can exert their emulsification and adsorption properties in emulsions and are improved by electrostatic or three-dimensional repulsive forces to maintain emulsion stability (30). CN, characterized by its disordered structure and hydrophobic properties, possesses greater adsorption at the oil–water interface than a tiny, compact, and globular WPI (31).

As represented in Figures 1B,C, K^+^ and Ca^2+^ binding may induce the aggregation of emulsion droplets as salt ions reduce the repulsive force between droplets (32). However, the particle size distribution peak of the samples shifted toward larger particle size with the addition of potassium ions, except for the ratio 5:5 in Figure 1B. According to Keowmaneechai et al., surfactants such as casein to replace the WP absorbed from the droplet surface, the aggregation induced by metal ions was found to be almost reversible (33). In addition, the homogenized proteins were exposed to more calcium binding sites, which might accelerate particle aggregation (34).

Therefore, compared with K^+^, emulsions with Ca^2+^ produce extensive droplet aggregation resulting in an unstable emulsion. The absolute value of the zeta potential was the highest when the proportion of CN was the highest, as shown in Figure 2. Amino acid side chains from protein dissociation are more conducive to induce electrostatic repulsion to enhance emulsion stability when the pH (the pH of emulsions is seven) is further away from the emulsifier's isoelectric point (35, 36). At pH values above the isoelectric point, protein molecules have more –COO^−^ groups than –NH3^+^ groups, so protein molecules present more negatively charged groups. It has been reported that Ca^2+^ can bind to the two main proteins of whey protein through the free carboxylic groups of aspartic and glutamic acids (35, 37). Therefore, Ca^2+^ could combine with the –COO^−^ groups to reduce the negative charge on the surface of emulsion droplets in this study. In the absence of K^+^, emulsions might lead to a less extensive diffuse double layer, interdroplet repulsion, and eventually droplet aggregation (38). The effect of ion valence on emulsion zeta potential in this study indicated that multivalent ions in the emulsion were more effective than monovalent ions combined with countercharge in the interaction effects of electrostatic shielding and ion surface adsorption. Our results were consistent with the conclusions of previous studies (39).

Creaming index is an important indicator to measure the degree of aggregation of lipid droplets in the emulsion. Lipid droplet network layout, structural rearrangement, and final phase separation kinetics are the critical aspects, which impact emulsion instability, and depletion flocculation is one of the main causes (40–42). Emulsions with a high creaming index are generally unstable (16). According to Srinivasan et al., a high CN emulsion content resulted in low stability due to depletion flocculation generated by the unabsorbed CN in the aqueous phase (43). Our results showed that the addition of K^+^ and Ca^2+^ plays an important role in emulsion stability. The electrostatic repulsion between the droplets was gradually shielded by the anti-ions around the droplets, and the small particulate matter adsorbed on each other, so that the large particles aggregated and the fat floated upward with the addition of K^+^ and Ca^2+^ (44). In addition, the effects of K^+^ and Ca^2+^ on creaming index might be gradually increasing particle size and making droplet aggregation to result in flocculation with storage days. A higher degree of flocculation is preferable to a higher degree of creaming in emulsions (45). Creaming instability measurements in the absence of K^+^ and Ca^2+^ support the result of particle size and zeta potential measurements.

The rheological property is also a key factor to consider in O/W emulsion stability. Previous research showed that the floccules created in the emulsion trapped water molecules in the two oil–water phases. This increased the effective volume fraction of particles and promoted the flocculated emulsion to have a higher viscosity than the unflocculated emulsion (45, 46). Our results indicated that the Ca^2+^- containing emulsion has a higher viscosity, as shown in Figure 3D. It would be expected that Ca^2+^ could promote the flocculation of droplets to a greater extent and increase its strength due to electrostatic screen interactions, reduce droplet charge, and act as ionic bridges, while potassium ion could only play a role of electrostatic screen interactions, with weak strength and low emulsion viscosity (33).

Emulsion destabilization is often due to various physical phenomena. As shown in Table 2, the emulsion particle size in each group increased to different degrees and the Ca^2+^ group had the highest particle size on day 40 of the storage period. Previous studies have indicated that flocculation, rather than coalescence, is the cause of droplet aggregation of emulsions with calcium (19) and potassium (47). Flocculation occurs because the metal ions reduce the electrostatic repulsion between the droplets, thus these droplets get closer. According to Ray and Rousseau, droplet aggregation caused by electrostatic action is blocked when the absolute value of the zeta potential of the emulsion is higher than 30 mV (58) (48). In contrast, the absolute value of the zeta potential of a calcium ion was 29.67 ± 0.40, which is the smallest among the three groups. This result showed that the emulsion of adding Ca^2+^ would be unstable, which was consistent with the previous results. The emulsion particle size and zeta potential during the 40 days of study indicated that the storage stability of each emulsion is different due to factors such as protein ratios and ionic strength, which is consistent with the results reported by Ye (49). The above results indicated that K^+^ and Ca^2+^ affect the long-term stability of OPO emulsions, which may have significant effects on the formulation of potassium- and calcium-enhanced emulsion-based products with a long expected shelf-life.

In addition to physical properties, lipid oxidation is a major issue in food storage and consumption, influencing the flavor, odor, and color of food (11). Our results showed that lipid oxidation might be caused by the different structures of WPI and CN, enabling their affinity for iron to be different. However, during the oxidation process, the principal product, hydroperoxide, dissociates into secondary oxidation products, such as malondialdehyde and hexanal, resulting in a decrease in the oxidation content of POV (50).

As shown in Figures 4B-b,C-c, our results indicated that both POV and TBARS values increase after adding K^+^ and Ca^2+^ into the emulsion, which can promote the oxidation of emulsions enabling emulsions to be unstable. The results were the same as particle size and creaming index.

We chose the different ratios of WPI and CN to better simulate the different stages of breastfeeding, and added OPO human milk fat substitute, which was conducive to the digestion and absorption of breast milk by infants. Furthermore, minerals are also essential, and K^+^ and Ca^2+^ account for a high proportion of minerals in infant formula. Thus, macronutrients and micronutrients complement each other. However, this infant formula was not ideal, which might be related to the concentration of added minerals. Therefore, the effect of varying mineral ion concentrations on the physicochemical properties of the OPO emulsion must be thoroughly explored to prepare a stable liquid infant formula. In addition, from a nutritional point of view, *in vitro* gastrointestinal digestion would be ideal, and could be further investigated.

## Conclusions

This study demonstrates the impact of protein ratios and mineral ions on the stability of OPO emulsions as mimics of a liquid infant formula. Changes in WPI to CN ratios had a significant impact on the zeta potential but not on particle size. The addition of Ca^2+^ increased the average particle size and decreased the zeta potential. It caused emulsion instability, possibly because Ca^2+^ reduced the charge of the droplets and acted as an ionic bridge by shielding the interaction between static electricity and ions. These interactions promoted flocculation of the droplets and subsequently failed to prevent oil oxidation.

## Data Availability Statement

The raw data supporting the conclusions of this article will be made available by the authors, without undue reservation.

## Author Contributions

QW finished the conceptualization, set the methodology, and wrote the original draft. YX finished the methodology and analyzed the data with the software. YL finished the investigation and validation. FQ guided the methodology and supervised the results. GM and XZ participated in project administration, acquired funding, and reviewed and edited this manuscript. All authors have read and accepted the published version of this manuscript.

## Conflict of Interest

The authors declare that the research was conducted in the absence of any commercial or financial relationships that could be construed as a potential conflict of interest.

## Publisher's Note

All claims expressed in this article are solely those of the authors and do not necessarily represent those of their affiliated organizations, or those of the publisher, the editors and the reviewers. Any product that may be evaluated in this article, or claim that may be made by its manufacturer, is not guaranteed or endorsed by the publisher.

## References

[1] Walker A. Breast milk as the gold standard for protective nutrients. J Pediatr. (2010) 156:S3–7. 10.1016/j.jpeds.2009.11.021 20105662

[2] He S Liu G Zhu X. Human breast milk-derived exosomes may help maintain intestinal epithelial barrier integrity. Pediatr Res. (2021) 90:366–72. 10.1038/s41390-021-01449-y 33731816

[3] Demmelmair H Koletzko B. Lipids in human milk. Best practice & research. Clin Endocrinol Metab. (2018) 32:57–68. 10.1016/j.beem.2017.11.002 29549961

[4] Qin XL Zhong JF Wang YH Yang B Lan DM Wang FH. 1,3-Dioleoyl-2-palmitoylglycerol-rich human milk fat substitutes: Production, purification, characterization and modeling of the formulation. Eur J Lipid Sci Technol. (2014) 116:282–90. 10.1002/ejlt.201300343

[5] Wei W Feng Y Zhang X Cao X Feng F. Synthesis of structured lipid 1,3-dioleoyl-2-palmitoylglycerol in both solvent and solvent-free system. LWT-Food Sci. Technol. (2015) 60:1187–94. 10.1016/j.lwt.2014.09.01310417216

[6] Wei W Jin Q Wang X. Human milk fat substitutes: past achievements and current trends. Prog Lipid Res. (2019) 74:69–86. 10.1016/j.plipres.2019.02.001 30796946

[7] Ilyasoglu H Gultekin-Ozguven M Ozcelik B. Production of human milk fat substitute with medium-chain fatty acids by lipase-catalyzed acidolysis: optimization by response surface methodology. LWT-Food Sci Technol. (2011) 44:999–1004. 10.1016/j.lwt.2010.11.027

[8] Carnielli VP Luijendijk I Vangoudoever JB Sulkers EJ Sauer P. Structural position and amount of palmitic acid in infant formulas: effects on fat, fatty acid, and mineral balance. Pediatr Res. (1994) 36:10–10. 10.1203/00006450-199407000-00047 8985844

[9] López-López A López-Sabater MC Campoy-Folgoso C Rivero-Urgell M Castellote-Bargalló AI. Fatty acid and sn-2 fatty acid composition in human milk from Granada (Spain) and in infant formulas. Eur J Clin Nutr. (2002) 56:1242–54. 10.1038/sj.ejcn.1601470 12494309

[10] Koo WW Hockman EM Dow M. Palm olein in the fat blend of infant formulas: effect on the intestinal absorption of calcium and fat, and bone mineralization. J Am Coll Nutr. (2006) 25:117–22. 10.1080/07315724.2006.10719521 16582027

[11] Ries D Ye A Haisman D Singh H. Antioxidant properties of caseins and whey proteins in model oil-in-water emulsions. Int Dairy J. (2010) 20:72–8. 10.1016/j.idairyj.2009.09.001

[12] Ji J Zhang J Chen J Wang Y Dong N Hu C. Preparation and stabilization of emulsions stabilized by mixed sodium caseinate and soy protein isolate. Food Hydrocoll. (2015) 51:156–65. 10.1016/j.foodhyd.2015.05.013

[13] Liang Y Gillies G Patel H Matia-Merino L Ye A Golding M. Physical stability, microstructure and rheology of sodium-caseinate-stabilized emulsions as influenced by protein concentration and non-adsorbing polysaccharides. Food Hydrocoll. (2014) 36:245–55. 10.1016/j.foodhyd.2013.10.006

[14] Yerramilli M Ghosh S. Long-term stability of sodium caseinate-stabilized nanoemulsions. J Food Sci Technol. (2017) 54:82–92. 10.1007/s13197-016-2438-y 28242906 PMC5305704

[15] Zembyla M Murray BS Radford SJ Sarkar A. Water-in-oil Pickering emulsions stabilized by an interfacial complex of water-insoluble polyphenol crystals and protein. J Colloid Interf Sci. (2019) 548:88–99. 10.1016/j.jcis.2019.04.010 30981966

[16] Abdolmaleki K Mohammadifar MA Mohammadi R Fadavi G Meybodi NM. The effect of pH and salt on the stability and physicochemical properties of oil-in-water emulsions prepared with gum tragacanth. Carbohydr Polym. (2016) 140:342–8. 10.1016/j.carbpol.2015.12.081 26876860

[17] Erxleben SW Pelan E Wolf B. Effect of ethanol on the stability of sodium caseinate stabilised emulsions. Food Hydrocoll. (2021) 121:107058. 10.1016/j.foodhyd.2021.107058

[18] Ye A Singh H. Interfacial composition and stability of sodium caseinate emulsions as influenced by calcium ions. Food Hydrocoll. (2001) 15:195–207. 10.1016/S0268-005X(00)00065-5

[19] Ye A Singh H. Influence of calcium chloride addition on the properties of emulsions stabilized by whey protein concentrate. Food Hydrocoll. (2000) 14:337–46. 10.1016/S0268-005X(00)00010-2

[20] Qian C Decker EA Xiao H McClements DJ. Physical and chemical stability of β-carotene-enriched nanoemulsions: Influence of pH, ionic strength, temperature, and emulsifier type. Food Chem. (2012) 132:1221–9. 10.1016/j.foodchem.2011.11.091 29243604

[21] Ravindran S Williams MAK Ward RL Gillies G. Understanding how the properties of whey protein stabilized emulsions depend on pH, ionic strength and calcium concentration, by mapping environmental conditions to zeta potential. Food Hydrocoll. (2018) 79:572–8. 10.1016/j.foodhyd.2017.12.003

[22] Owens C Griffin K Khouryieh H Williams K. Creaming and oxidative stability of fish oil-in-water emulsions stabilized by whey protein-xanthan-locust bean complexes: impact of pH. Food Chem. (2018) 239:314–22. 10.1016/j.foodchem.2017.06.096 28873574

[23] Castel V Rubiolo AC Carrara CR. Droplet size distribution, rheological behavior and stability of corn oil emulsions stabilized by a novel hydrocolloid (Brea gum) compared with gum arabic. Food Hydrocoll. (2017) 63:170–7. 10.1016/j.foodhyd.2016.08.039

[24] Qiu C Zhao M Decker EA McClements DJ. Influence of protein type on oxidation and digestibility of fish oil-in-water emulsions: gliadin, caseinate, and whey protein. Food Chem. (2015) 175:249–57. 10.1016/j.foodchem.2014.11.112 25577077

[25] Zeng T Wu ZL Zhu JY Yin SW Tang CH Wu LY. Development of antioxidant Pickering high internal phase emulsions (HIPEs) stabilized by protein/polysaccharide hybrid particles as potential alternative for PHOs. Food Chem. (2017) 231:122–30. 10.1016/j.foodchem.2017.03.116 28449988

[26] Xu X Liu W Luo L Liu C McClements DJ. Influence of anionic polysaccharides on the physical and oxidative stability of hydrolyzed rice glutelin emulsions: impact of polysaccharide type and pH. Food Hydrocoll. (2017) 72:185–94. 10.1016/j.foodhyd.2017.05.018

[27] Dickinson E. Introduction to food colloids. J Study Relig Ideol. (1992) 64:22–23.

[28] Guzun-Cojocaru T Koev C Yordanov M Karbowiak T Cases E Cayot P. Oxidative stability of oil-in-water emulsions containing iron chelates: Transfer of iron from chelates to milk proteins at interface. Food Chem. (2011) 125:326–33. 10.1016/j.foodchem.2010.08.004

[29] Sugiarto M Ye A Singh H. Characterisation of binding of iron to sodium caseinate and whey protein isolate. Food Chem. (2009) 114:1007–13. 10.1016/j.foodchem.2008.10.062

[30] Kellerby SS McClements DJ Decker EA. Role of proteins in oil-in-water emulsions on the stability of lipid hydroperoxides. J Agric Food Chem. (2006) 54:7879–84. 10.1021/jf061340s 17002465

[31] Hu M McClements DJ Decker EA. Impact of whey protein emulsifiers on the oxidative stability of salmon oil-in-water emulsions. J Agric Food Chem. (2003) 51:1435–9. 10.1021/jf0203794 12590494

[32] Shao Y Tang CH. Characteristics and oxidative stability of soy protein-stabilized oil-in-water emulsions: influence of ionic strength and heat pretreatment. Food Hydrocoll. (2014) 37:149–58. 10.1016/j.foodhyd.2013.10.030

[33] Keowmaneechai E McClements DJ. Effect of CaCl2 and KCl on physiochemical properties of model nutritional beverages based on whey protein stabilized oil-in-water emulsions. J Food Sci. (2002) 67:665–671. 10.1111/j.1365-2621.2002.tb10657.x 12428974

[34] Radford SJ Dickinson E Golding M. Stability and rheology of emulsions containing sodium caseinate: combined effects of ionic calcium and alcohol. J Colloid Interface Sci. (2004) 274:673–86. 10.1016/j.jcis.2003.12.045 15144844

[35] Pappas CP Rothwell J. The effects of heating, alone or in the presence of calcium or lactose, on calcium binding to milk proteins. Food Chem. (1991) 42:183–201. 10.1016/0308-8146(91)90033-K

[36] Walker HW Grant SB. Influence of surface charge and particle size on the stabilization of colloidal particles by model polyelectrolytes. Coll Surf A. (1998) 135:123–33. 10.1016/S0927-7757(97)00226-4

[37] Baumy JJ Brule G. Binding of bivalent cations to a-lactalbumin and b-lactoglobulin: effect of pH and ionic strength. Lait. (1988) 68:33–48. 10.1051/lait:198813

[38] Kulmyrzaev AA Schubert H. Influence of KCl on the physicochemical properties of whey protein stabilized emulsions. Food Hydrocoll. (2004) 18:13–9. 10.1016/S0268-005X(03)00037-7

[39] Shao P Ma H Zhu J Qiu Q. Impact of ionic strength on physicochemical stability of o/w emulsions stabilized by Ulva fasciata polysaccharide. Food Hydrocoll. (2017) 69:202–9. 10.1016/j.foodhyd.2017.01.039

[40] Cuevas-Bernardino JC Lobato-Calleros C Román-Guerrero A Alvarez-Ramirez J Vernon-Carter EJ. Physicochemical characterisation of hawthorn pectins and their performing in stabilising oil-in-water emulsions. React Funct Polym. (2016) 103:63–71. 10.1016/j.reactfunctpolym.2016.03.024

[41] Hu HY Xing LJ Hu YY Qiao CL Wu T Zhou GH. Effects of regenerated cellulose on oil-in-water emulsions stabilized by sodium caseinate. Food Hydrocoll. (2016) 52:38–46. 10.1016/j.foodhyd.2015.06.019

[42] Liang Y Gillies G Matia-Merino L Ye A Patel H Golding M. Structure and stability of sodium-caseinate-stabilized oil-in-water emulsions as influenced by heat treatment. Food Hydrocoll. (2016) 66:307–17. 10.1016/j.foodhyd.2016.11.041

[43] Srinivasan M Singh H Munro PA. The effect of sodium chloride on the formation and stability of sodium caseinate emulsions. Food Hydrocoll. (2000) 14:497–507. 10.1016/S0268-005X(00)00030-8

[44] Xiao J Wang X Gonzalez AP Huang QR. Kafirin nanoparticlesstabilized Pickering emulsions: Microstructure and rheological properties. Food Hydrocoll. (2016) 54:30e3943. 10.1016/j.foodhyd.2015.09.008

[45] Sriprablom J Luangpituksa P Wongkongkatep J Pongtharangkul T Suphantharika M. Influence of pH and ionic strength on the physical and rheological properties and stability of whey protein stabilized o/w emulsions containing xanthan gum. J Food Eng. (2019) 242:141–52. 10.1016/j.jfoodeng.2018.08.031

[46] Dickinson E. Properties of emulsions stabilized with milk proteins: overview of some recent developments. J Dairy Sci. (1997) 80:2607–19. 10.3168/jds.S0022-0302(97)76218-0

[47] Hunt JA Dalgleish DG. The effect of the presence of KCl on the adsorption behavior of whey protein and caseinate in oil-in-water emulsions. Food Hydrocoll. (1996) 10:159–65. 10.1016/S0268-005X(96)80030-0

[48] Ray M Rousseau D. Stabilization of oil-in-water emulsions using mixtures of denatured soy whey proteins and soluble soybean polysaccharides. Food Res Int. (2013) 52:298–307. 10.1016/j.foodres.2013.03.008

[49] Ye A. Interfacial composition and stability of emulsions made with mixtures of commercial sodium caseinate and whey protein concentrate. Food Chem. (2008) 110:946–52. 10.1016/j.foodchem.2008.02.091 26047284

[50] Chen B McClements DJ Decker EA. Role of continuous phase anionic polysaccharides on the oxidative stability of menhaden oil-in-water emulsions. J Agric Food Chem. (2010) 58:3779–84. 10.1021/jf9037166 20158199

